# Lipofuscin-Mediated Photic Stress Induces a Dark Toxic Effect on ARPE-19 Cells

**DOI:** 10.3390/ijms232012234

**Published:** 2022-10-13

**Authors:** Tatiana Feldman, Dmitriy Ostrovskiy, Marina Yakovleva, Alexander Dontsov, Sergey Borzenok, Mikhail Ostrovsky

**Affiliations:** 1 Department of Biology, Lomonosov Moscow State University, Leninskiye Gory 1, 119234 Moscow, Russia; 2 Emanuel Institute of Biochemical Physics, Russian Academy of Sciences, 4 Kosygin Street, 119334 Moscow, Russia; 3 Koltzov Institute of Developmental Biology, Russian Academy of Sciences, 26 Vavilov Street, 119334 Moscow, Russia; 4 Sv. Fyodorov Eye Microsurgery Complex, 59a Beskudnikovsky bld., 127486 Moscow, Russia

**Keywords:** age-related macular degeneration, retinal pigment epithelium, lipofuscin granules, bisretinoid fluorophores, bisretinoid oxidation and degradation products, cytotoxicity

## Abstract

Lipofuscin granules from retinal pigment epithelium (RPE) cells contain bisretinoid fluorophores, which are photosensitizers and are phototoxic to cells. In the presence of oxygen, bisretinoids are oxidized to form various products, containing aldehydes and ketones, which are also potentially cytotoxic. In a prior study, we identified that bisretinoid oxidation and degradation products have both hydrophilic and amphiphilic properties, allowing their diffusion through the lipofuscin granule membrane into the RPE cell cytoplasm, and are thiobarbituric acid (TBA)-active. The purpose of the present study was to determine if these products exhibit a toxic effect to the RPE cell also in the absence of light. The experiments were performed using the lipofuscin-fed ARPE-19 cell culture. The RPE cell viability analysis was performed with the use of flow cytofluorimetry and laser scanning confocal microscopy. The results obtained indicated that the cell viability of the lipofuscin-fed ARPE-19 sample was clearly reduced not immediately after visible light irradiation for 18 h, but after 4 days maintaining in the dark. Consequently, we could conclude that bisretinoid oxidation products have a damaging effect on the RPE cell in the dark and can be considered as an aggravating factor in age-related macular degeneration progression.

## 1. Introduction

The neural retina is considered to be an extension of the central nervous system [1] and has several features in common with the brain. The neural retina is similarly sensitive to degenerative processes, in which the closely adjacent retinal pigment epithelium (RPE) plays a key role. There are various forms of degenerative processes, including one of the leading causes of visual impairment, age-related macular degeneration (AMD). The molecular mechanisms of AMD are continuously being elucidated, with the focus on oxidative stress [2,3,4,5].

The neural retina is the light-sensitive tissue of the eye. It consists of several layers of neurons interconnected by synapses. The primary light-sensing cells in the retina are photoreceptor cells, rods, and cones. The RPE is the pigmented single-cell layer located right behind the retina, firmly attached to the underlying choroid and in close contact with photoreceptor cells. The RPE has several crucial functions for vision, namely, scattered light absorption, epithelial transport, spatial ion buffering, visual cycle, phagocytosis of outer segment photoreceptor membranes, secretion, and immune modulation [6].

With exposure to light, during rhodopsin photolysis, toxic retinoid side products can be produced in photoreceptor cells. Biogenesis of these products occurs when two molecules of all-*trans* retinal condense with one molecule of phosphatidylethanolamine in the photoreceptor membrane [7]. Evolution has developed a powerful mechanism that prevents the accumulation of retinoid side products in terminally differentiated photoreceptor cells [8]. Throughout life, the debris of the photoreceptor outer segment (POS) apical part is phagocytized and digested by RPE cells, while new photoreceptor discs with rhodopsin molecules are synthesized by the photoreceptor inner segments [9]. However, the lysosomal enzyme system of the RPE cell is not effective in the degrading of POS debris, because the latter is supposed to contain modified retinoid side products of rhodopsin photolysis, as well as modified lipids and proteins. In other words, the lysosomal enzyme system of the RPE cell cannot recognize such modified molecules and do not digest them [10]. As a consequence, lipofuscin granules (LGs), containing retinoid derivatives, are formed and accumulated in RPE cells with age [11,12,13]. They have been long believed to be just a cell metabolism by-product. However, it has been established that LGs are one of the sources of reactive oxygen species (ROS) in RPE cells. Visible light exposure induces ROS formation in LGs, initiating oxidative stress in RPE cells [14]. Oxidative stress is central to the development of AMD [2,3,4,5]. It is characterized by increased level of ROS resulting in damage or modification of cellular proteins, lipids, and DNA, impairing their physiological functions. Therefore, the development of AMD is associated with the progressive accumulation of LGs in the RPE [9]. LGs are, therefore, a risk factor for degenerative processes in the retina and RPE [15,16,17].

The main photoinducible generators of ROS in LGs are retinoid side products bisretinoids (BisRets) and their oxidation and degradation derivatives (BisRets-OX) [18,19,20,21,22,23,24,25]. *N*-retinyl-*N*-retinylidenethanolamine (A2E) is the most widely studied BisRet [18,19,20]. Retinoid side products are major sources of LG fluorescence [9,21]. The features of BisRets, such as photosensitization, have been studied in detail [14,26,27,28,29,30]. 

It should be noted that BisRets themselves can be photooxidized to form various products, consisting primarily of aldehydes and ketones [23,25,31,32,33]. It is known that reactive carbonyls are highly cytotoxic; they can modify cellular proteins and lipids and can cause carbonyl stress [34,35]. BisRets-OX with increased hydrophilicity were previously thought to leave LGs and enter the RPE cell cytoplasm [36], directly damaging the cell structure [37].

Our prior in vitro experiments [38] have demonstrated that BisRets-OX have hydrophilic and amphiphilic properties and are thiobarbituric acid (TBA)-active. Therefore, almost all detectable BisRet-OX species can potentially be released from LGs. Thus, they can damage RPE cellular organelles and macromolecules. Since carbonyl products are long-lived, they can tightly bind with long-lived proteins such as collagen [39] or hemoglobin [40], resulting in the formation of advanced glycation end products that can activate inflammatory processes. The results of our in vitro experiments [41] have shown that water-soluble carbonyl products formed by A2E photodestruction lead to the formation of modified proteins, which also indicates that BisRets-OX can damage RPE cells.

Therefore, despite the fact that BisRet-OX species absorb in the short-wavelength region of the spectrum (UV) and cannot be photoactive in vivo, they can nevertheless be toxic to RPE cells. Thus, formation of BisRet-OX-containing active carbonyls, which accumulate in LGs of the RPE cell, may play an important role in the pathogenesis of eye diseases, including AMD.

The present research aimed to study the dark toxic effect of LGs on RPE cells after irradiation with visible light when the level of BisRet-OX-containing active carbonyls was increased [38]. The experiments were performed using LGs obtained from human cadaveric eyes without pathology and the ARPE-19 cell culture.

## 2. Results

### 2.1. Characteristics of Lipofuscin-Fed RPE Cell Samples

**Figure 1:**
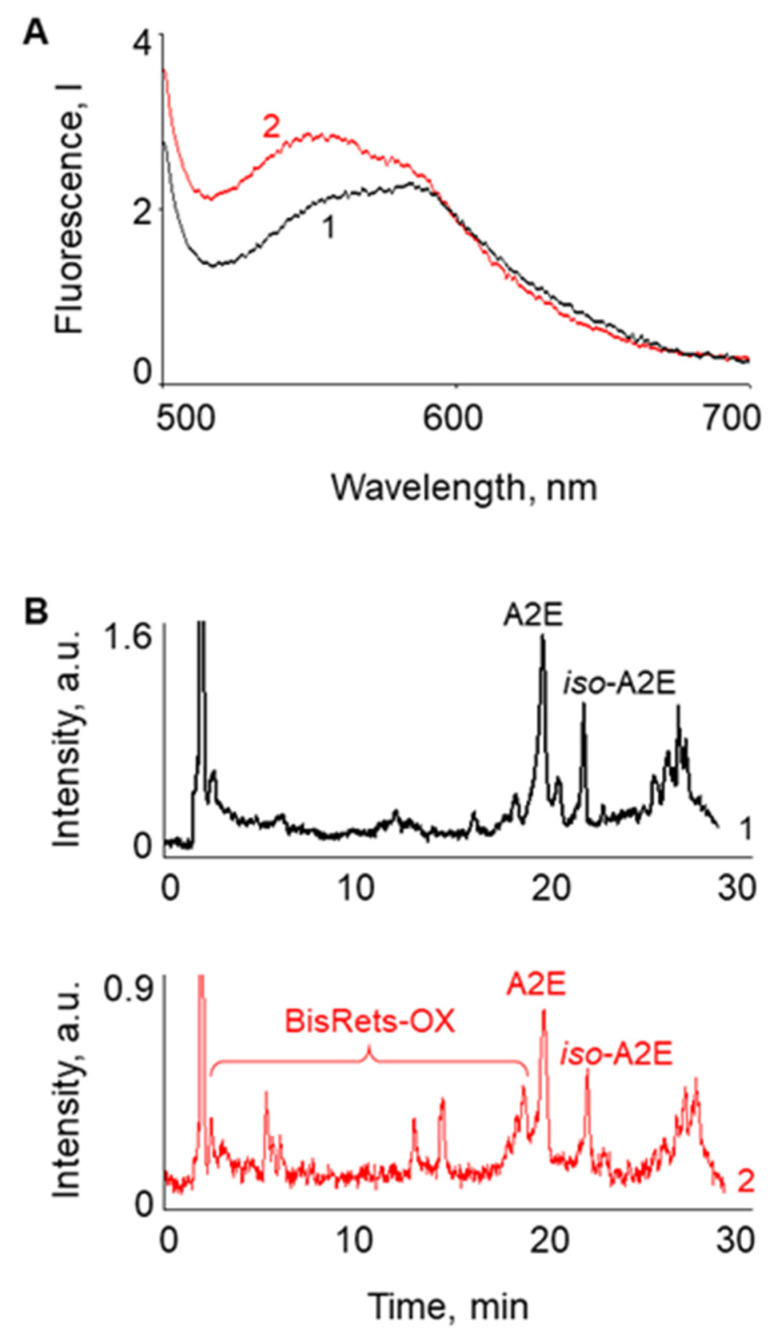
(**A**) Fluorescence spectra of lipofuscin granule (LG) suspensions: 1—native nonirradiated; 2—irradiated with visible light for 10 min. The wavelength of fluorescence excitation was 488 nm; spectra were normalized to the wavelength of 592 nm. (**B**) HPLC analysis of LG chloroform extracts: 1—from native nonirradiated LGs; 2—from LGs irradiated with visible light for 10 min. Detection was performed at the wavelength of 430 nm.

**Figure 2:**
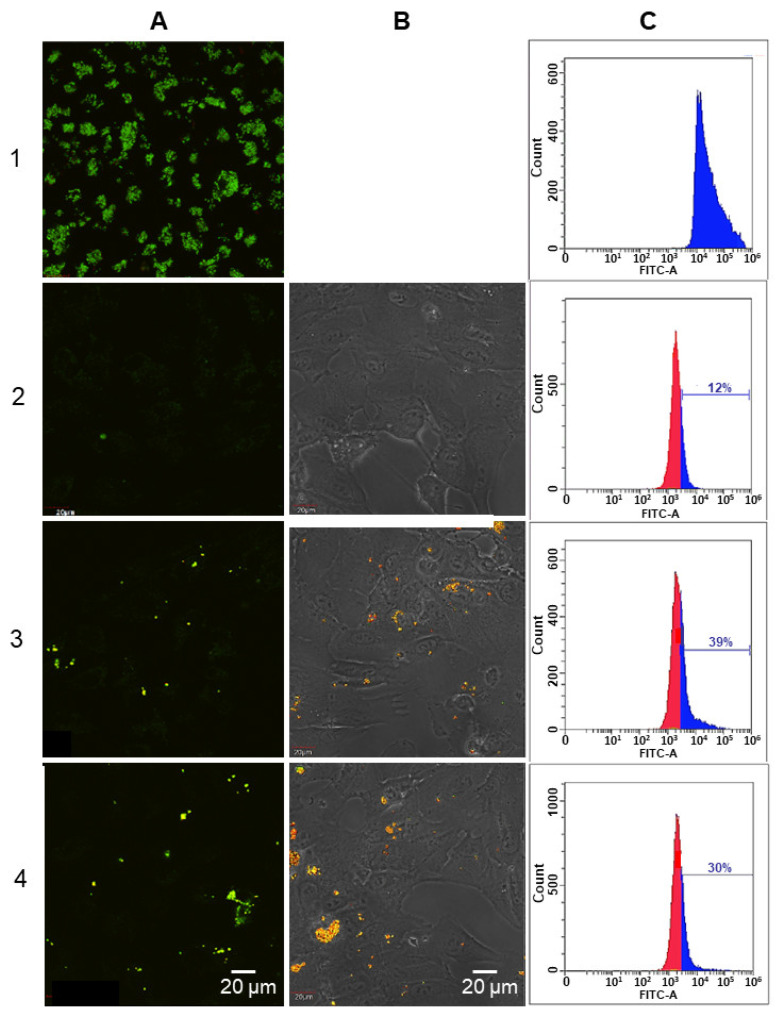
Laser scanning confocal microscopy: (**A**) fluorescence using the green channel AF 488 (Ex 490/Em 525); (**B**) phase contrast simultaneously with the image of the lipofuscin-fed RPE cell fluorescence, magnification: 600×. Flow cytofluorimetry (**C**) using detection channels such as FITC (Ex 493/Em 528) and Cy3 (Ex 550/Em 615): on the abscissa axis, fluorescence intensity is presented; on the ordinate axis, the values correspond to the number of RPE cells analyzed. Line 1—original lipofuscin granule (LG) suspension; 2—original RPE cell culture without LGs; 3—LG-fed RPE cells; 4—LG*-fed RPE cells.

Two types of LGs were used to conduct the experiment. The LG suspension obtained from cadaveric eye RPEs was divided into two equal parts. One sample was preirradiated with visible light and the other was used in its original native state. The samples were irradiated in order to obtain an increased content of BisRets-OX in LGs. Previously, we found that an increased content of BisRets-OX was observed in LGs obtained from the RPE of cadaveric eyes with AMD pathology [25,42]. This difference was manifested in the spectral properties of LG suspensions and the results of a HPLC analysis of chloroform extracts from these suspensions. Figure 1A demonstrates the fluorescence spectra of an unirradiated (1) and visible light-irradiated (2) LG suspension. An increase in the fluorescence intensity of irradiated LGs in the short-wavelength part of the spectrum (530–580 nm) indicated an increase in the content of BisRets-OX [25,43,44]. A HPLC analysis also confirmed an increase in oxidized products [25,43] in the irradiated LG sample (Figure 1B and Figure 2).

Two samples of lipofuscin-fed RPE cells were obtained. The first sample contained original LGs (subsequently referred to as LG-fed RPE) and the other contained LGs irradiated with visible light (subsequently referred to as LG*-fed RPE). The sample of RPE cells without LGs was used as a control sample. 

Laser scanning confocal microscopy (qualitative analysis) revealed the presence of cells with fluorescence in the RPE samples (Figure 2A,B). Flow cytofluorimetry (quantitative analysis) demonstrated that the LG-fed RPE and LG*-fed RPE samples contained approximately 39% and 30% of cells, respectively, exhibiting fluorescent properties (Figure 2C, lines 3 and 4). 

It should be noted that in the control sample (Figure 2, line 2), approximately 12% of the cells also exhibited fluorescent properties. However, the HPLC analysis showed that BisRets or BisRets-OX were not the source of this fluorescence. It could be assumed that this fluorescence was caused by other components of the cell, including FADs (flavinadenindinucleotides) and AGEs (advanced glycation end products), that have weak fluorescence in the same spectral area as LGs [45]. Thus, taking into account the fluorescence of an unidentified source, it could be concluded that LG-fed RPE and LG*-fed RPE samples contained phagocytized original LGs or visible light-irradiated ones, respectively.

### 2.2. Toxic Effect of Lipofuscin Granules on the RPE Cells

In earlier in vitro experiments [38], we demonstrated that BisRets-OX, which are formed when LGs are irradiated with visible light, have both hydrophilic and amphiphilic properties, allowing their diffusion through the LG membrane into the RPE cell cytoplasm. It was established that these products contain cytotoxic carbonyls, which can modify cellular proteins and lipids [41].

In this work, each sample was divided into two equal parts. Thus, two groups of samples were obtained, each consisting of a control sample (RPE cells without LGs), LG-fed RPE sample, and LG*-fed RPE sample. The first group of samples was maintained in the dark. The second group of samples was irradiated with visible light for 18 h. Subsequent analyses were performed simultaneously for both groups.

**Figure 3:**
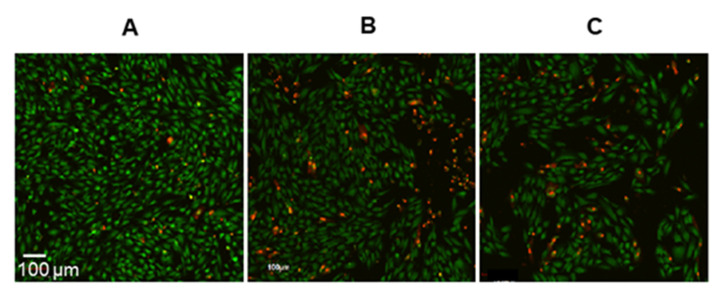
Confocal microscope images of RPE cells stained with fluorescent dye Live and Dead. The green channel AF 488 (Ex 490 nm/Em 525 nm) and red channel AF 594 (Ex 590 nm/Em 617 nm) were used. Magnification: 100×. Living cells are green and dead cells are red. (**A**) Control RPE cell sample without lipofuscin granules (LGs). (**B**) LG-fed RPE sample. (**C**) LG*-fed RPE sample. The images were obtained after the samples were irradiated with visible light for 18 h and then maintained in the dark for 4 days.

To analyze RPE cell viability, the samples were stained with the fluorescent dye Live and Dead. Figure 3 demonstrates the qualitative confocal microscope images of RPE cells after irradiation of samples from the second group with visible light and subsequent maintenance in the dark for 4 days.

**Figure 4:**
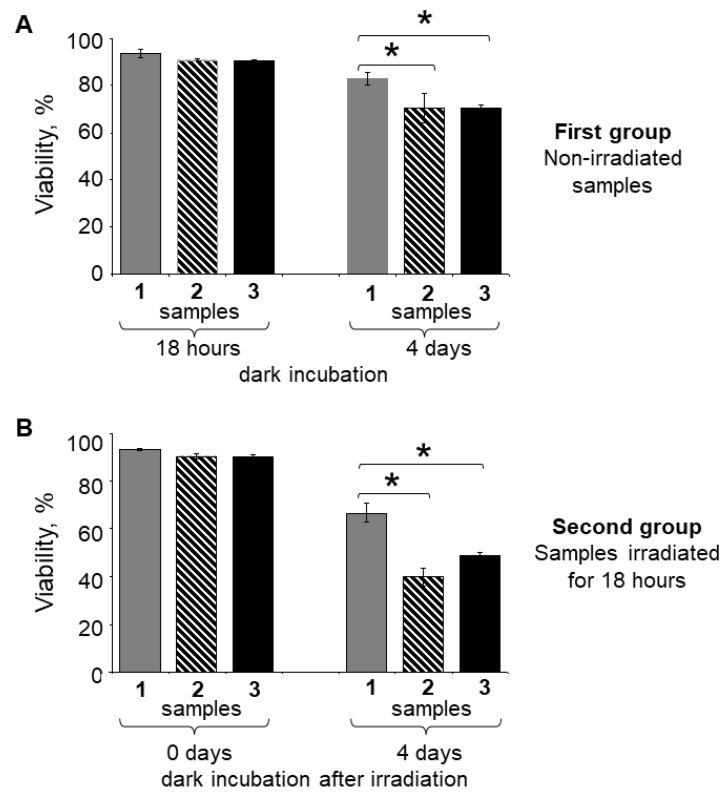
Comparative flow cytofluorimetry analysis of the cell viability. RPE cell samples were stained with fluorescent dye Live and Dead. The detection channels FITC (Ex 493 nm/Em 528 nm) and Cy3 (Ex 550 nm/Em 615 nm) were used. Samples: 1—original RPE cell culture without lipofuscin granules (LGs); 2—LG-fed RPE cells; 3—LG*-fed RPE cells. (**A**) All samples were maintained in the dark for 18 h, and then 4 days. (**B**) All samples were irradiated with visible light for 18 h, and then maintained in the dark for 4 days. Data are presented as means ± SDs from three independent experiments (2 replicates per experiment). * Values of *p* < 0.05 were considered significant.

A quantitative analysis of RPE cell viability was performed using flow cytofluorimetry (Figure 4). The subsequent comparative analysis of two groups of samples, where the first group was in dark conditions for 18 h and the second one was irradiated with visible light for the same amount of time, showed that there were no significant changes in the content of living RPE cells in all samples (Figure 4A,B). Approximately 93% of living RPE cells were detected in control samples (1), both dark-adapted (A) and irradiated with visible light (B), and approximately 90% of living RPE cells were detected in LG-fed RPE (2) and LG*-fed RPE (3) samples in both experimental conditions (Table 1).

The following incubation of the samples of both groups in the dark for 4 days led to the death of some RPE cells in all samples (Figure 4A,B; Table 1). However, it should be noted that the degree of cell death in nonirradiated samples and those irradiated with visible light for 18 h was different. In the case of nonirradiated samples, the degree of cell death was lower compared to irradiated ones (Table 1). This fact demonstrated the damaging effect of visible light both in lipofuscin-fed RPE cells and in unfed RPE cells. At the same time, the degree of lipofuscin-fed RPE cell death was higher compared to unfed RPE cells for both maintained-in-the-dark samples and irradiated samples with subsequent dark adaptation for 4 days (Figure 4A,B; Table 1).

The difference in the degree of death between maintained-in-the-dark samples 2 and 3 (lipofuscin-fed RPE cells) was not significant. Therefore, we could conclude that the effect of light exposure was the same for both LG-fed RPE cells and LG*-fed RPE cells.

It should be noted that the consequences of destructive processes in the RPE cells began to manifest themselves noticeably only after dark incubation for 4 days. This may indicate that additional dark processes initiated by light played an important role in the mechanism of photosensitized damage to the RPE cell. 

Thus, this experiment showed that a significant cytotoxic effect was observed not immediately after irradiation of the samples, but after some time. This suggests that, indeed, for cytotoxic hydrophilic and amphiphilic BisRets-OX [38], time is needed for the diffusion from LGs into the cytoplasm of the RPE cell and the manifestation of a damaging effect on cell structures. 

### 2.3. MTT Cell Viability Assay 

Our findings that BisRets-OX are TBA-active and have the potential to release LGs [38] allowed us to assume that they can damage RPE cellular organelles and macromolecules in the dark [41]. 

The MTT (3-(4,5-dimethylthiazol-2-yl)-2,5-diphenyl tetrazolium bromide) assay was used as an additional indicator of RPE cell viability [46,47]. The MTT assay was based on the ability of a mitochondrial dehydrogenase enzyme from a viable cell to cleave the tetrazolium rings of the pale-yellow MTT and form dark-blue formazan crystals that are largely impermeable to cell membranes, thus, resulting in its accumulation within healthy cells. Dead cells were unable to cleave MTT. 

**Figure 5:**
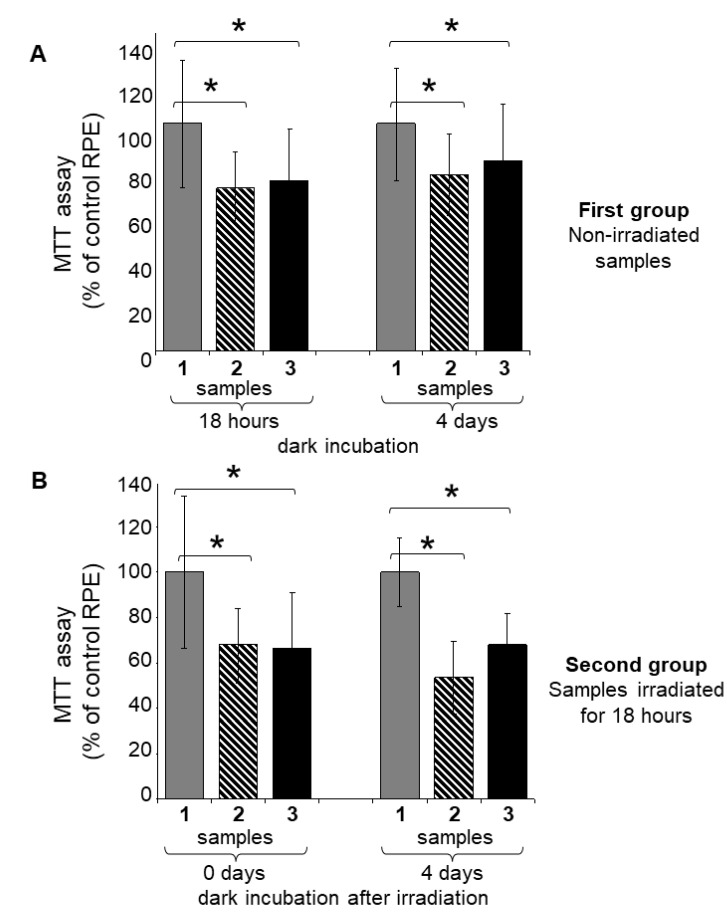
MTT cell viability assay. Samples: 1—original RPE cell culture without lipofuscin granules (LGs); 2—LG-fed RPE cells; 3—LG*-fed RPE cells. (**A**) All samples (first group) were maintained in the dark for 18 h, and then 4 days. (**B**) All samples (second group) were irradiated with visible light for 18 h, and then maintained in the dark for 4 days. Data are presented as means ± SDs from three independent experiments (20 replicates per experiment). * Values of *p* < 0.05 were considered significant.

The cell viability was assessed as the intensity of the product color, which was directly proportional to the number of living cells in the RPE culture (Figure 5). The MTT assay showed that in all cases, regardless of the experimental conditions, the relative level of living cells was noticeably lower in the lipofuscin-fed RPE samples compared to the control RPE without LGs (Figure 5; Table 2). 

At the same time, in the first group of samples maintained in the dark, the level of cell viability was higher compared to irradiated samples (Table 2). At that time, in the first group of samples (Figure 5A), there was a slight increase in cell viability in lipofuscin-fed RPE samples (on average from 73% to 80%) during dark incubation (Table 2). In contrast, no such growth was observed in the second group of samples (Figure 5B) exposed to visible light. Moreover, in the lipofuscin-fed RPE samples maintained in the dark after irradiation, there was a significant (*p* < 0.05) decrease in cell viability (on average from 68% to 54%) (Figure 5B; Table 2). Thus, in all experiments, both lipofuscin-fed cells maintained in the dark and irradiated ones showed a loss of viability compared with unfed RPE cells. At the same time, in the case of lipofuscin-fed cells exposed to light, the greatest loss of viability was observed. As in the previous experiment (Figure 4B), there were no significant differences between lipofuscin-fed RPE cell samples maintained in the dark and irradiated samples. 

The MTT assay data correlated with the results of the RPE cell viability analysis with flow cytofluorimetry using the fluorescent dye Live and Dead. However, it should be noted that in the case of the MTT analysis, the values of cell viability for lipofuscin-fed RPE samples differed markedly from the control when assessing cell viability of samples maintained in the dark (Figure 4A and Figure 5A; 18 h) and immediately after irradiation with visible light (Figure 4B and Figure 5B; 0 days). This difference can be explained by the fact that MTT itself can disrupt the work of mitochondria during the study of the mitochondrial dehydrogenase activity [48]. 

Thus, the greatest cytotoxicity in relation to RPE cells was shown by lipofuscin-fed RPE samples maintained in the dark after irradiation. These results support our assumption [38,41] that BisRets-OX with hydrophilic and amphiphilic features can have cytotoxic effects on RPE cells. The results of our experiments correlate with the other literature data [47,49,50]. 

## 3. Discussion

It is known that the exposure of LG BisRets to visible light leads to their oxidation and degradation. The cytotoxic properties of BisRets-OX in LGs have not been fully investigated. The role of these compounds in pathological processes of the RPE remains controversial. Some studies have suggested that highly reactive cytotoxic carbonyl compounds, aldehydes and ketones, are formed during the photooxidation of BisRets in LGs [22,26,51]. By contrast, other studies [52,53] have suggested BisRets-OX interact with themselves or with A2E, forming products with a higher molecular weight inside LGs. Most of these compounds are also hydrophobic and remain inside LGs, resulting in the concomitant diminution in its reactivity in vivo [52,53].

Clarifying the role of BisRets-OX is important to delineate the mechanisms of pathological ocular diseases, especially AMD. Our prior findings [42] have demonstrated that LG BisRet-OX content is higher in AMD eyes than in normal eyes, which was indicated by changes in the characteristics of LG fluorescence spectra and in the parameters of fluorescence decay kinetic curves. Specifically, the fluorescence intensity (excitation at 488 nm) of samples from eyes with AMD increased in the range of 556 nm, and the contribution of BisRets-OX to total fluorescence rose. However, the pathophysiological or protective properties of these products remain controversial, as prior studies have suggested conflicting roles [52,53], and as BisRets-OX could also potentially become a neutral product eventually.

Our in vitro experiments [38,41] have shown that BisRets-OX consist of aldehydes and ketones which are accumulated in the free state within LGs. It has also been demonstrated that these substances have hydrophilic and amphiphilic properties capable of diffusing from LGs into the cytoplasmic part of the RPE cell. Since they are highly reactive products, it has been proposed that these BisRets-OX can have a toxic effect on the RPE cell, independent of light [38,41]. Moreover, Zhou et al. [54] demonstrated that the complex mixture of products formed during photooxidation of the lipofuscin fluorophore A2E can activate the complement cascade, leading to the development of inflammatory processes. It is known that chronic inflammation contributes to the pathogenesis of many senile diseases [55], including the development of “dry” and “wet” forms of AMD [56,57,58,59,60]. However, the triggers responsible for the activation of local inflammatory responses in RPE cells remain unknown. 

It has been suggested [41,54] that one of the possible triggers of inflammation could be the photooxidation products of the RPE lipofuscin. These products cause protein modification, resulting in the accumulation of defective proteins that can be recognized by the complement system as “foreign”. It can be assumed that functionally important proteins, when modified with water-soluble A2E photooxidative destruction products, can activate the complement system, initiate inflammation, and lead to the development of AMD upon prolonged exposure to cells. Hence, serum albumin and hemoglobin beta 2 were identified in the drusen composition obtained from donor eyes of healthy and AMD patients, with a significantly higher concentration in AMD patients [61,62]. Thus, the interaction of proteins with water-soluble products of A2E photooxidative destruction leads to the accumulation of modified damaged proteins. The accumulation of such modified proteins can lead to the development of inflammatory processes and, importantly, underlie the pathogenesis of many eye diseases, including senile diseases in which the bisretinoid A2E accumulates in RPE cells.

In this work, the study of the BisRet-OX toxic effect on the RPE cell was performed. A comparative analysis of the degree of RPE cell death after irradiation with visible light showed that the death level was higher in samples containing LGs compared to RPE cells without LGs. At the same time, the effect was largely manifested not immediately, but after sample dark incubation for 4 days. In addition, a similar effect was observed in the case of the MTT assay. At the same time, it should be noted that the contribution of the cellular protein photooxidation process [63] to cell death could not be excluded.

Thus, the results allowed us to believe that LG BisRets can not only be phototoxic [14,26,27,28,29,30], but also exhibit toxic features against RPE cellular biomolecules and structures as a result of their oxidation and degradation. It should be noted that BisRets can be oxidized not only as a result of exposure to light in the presence of oxygen, but also due to ROS of a “nonlipofuscin” nature. We have demonstrated this phenomenon in in vitro and in vivo experiments [38,64]. In the study by Yakovleva et al. [38], BisRet-OX formation was observed after exposure of LG suspension to superoxide radicals. In experiments in vivo [64], ionizing radiation (IR)-mediated retinoid oxidation in the retina and RPE was analyzed. IR is known to prompt the generation of ROS and subsequent oxidative stress [65,66]. A comparative fluorescence and chromatographic analysis of retinoids before and after IR showed that the fluorescent properties of chloroform extracts from irradiated mouse retina and the RPE exhibited an increase in fluorescence intensity in the short-wave region of the spectrum (λ < 550 nm). This change was due to increased retinal and RPE retinoid oxidation and degradation products after IR exposure.

A growing body of evidence from cell culture and animal models suggests that oxidative stress is central in the development of AMD due to its relationship with other molecular mechanisms of AMD [2,3]. Oxidative stress drives functional RPE alterations, such as the oxidation of proteins and lipids, and induces mitochondrial DNA damage [63,67,68]. Hence, Olchawa et al. [68] demonstrated that mild oxidative stress, mediated by the age pigment lipofuscin, impairs specific phagocytic activity of the RPE. Wiktor et al. [63] found that sublethal or weekly lethal photic stress, mediated by phagocytized human RPE LGs, affects nanomechanical properties of ARPE-19 cells. They showed that oxidative stress, accompanying photic stress, leads to the oxidation of cellular proteins and the disruption of the cell cytoskeleton. 

Therefore, ROS of any nature can oxidize BisRets, leading to the formation of reactive carbonyls in the free state [38]. Reactive carbonyls are highly cytotoxic and can cause carbonyl stress [34,35]. As we have previously shown [38,41], some of these compounds, having hydrophilic and amphiphilic properties, can diffuse through the LG membrane into the cytoplasm of the RPE cell. It is important to note that these carbonyl products contain water-soluble amphiphilic compounds that can damage not only the water-soluble proteins of the cytoplasm, but also various membrane structures of the cell. Protein modification caused by these substances can activate inflammatory processes, and, thus, is of great importance in the pathogenesis of many eye diseases, including AMD.

It should be noted that the effect of the BisRet-OX impact was not detected immediately, but after a while. This can be explained by the fact that it took time for the process of diffusion of BisRets-OX from the LG to the RPE cell. Thus, the results of this work also support the assumption about the cytotoxic properties of BisRets-OX. The results correlate well with the data of other researchers [61,69,70]. It has been shown that in RPE cells from donor eyes with AMD, compared with normal eyes, there are increased levels of TBA-active products and protein carbonyls [69], and a high content of carboxyethylpyrrole in Bruch’s membrane [61,70].

## 4. Materials and Methods

### 4.1. Tissues and Reagents

Experiments on human cadaver eye tissues were performed in compliance with officially accepted procedures, specifically the Russian Federation law N 4180-I dated 22 December 1992, “On human organs or tissue transplantation” (with the most recent modifications and additions dated 8 December 2020). Human cadaver eyes without ophthalmologic disease were obtained from the Eye Tissue Bank of the S.N. Fyodorov Eye Microsurgery Complex (Moscow, Russia) within 10 h of donor death. Donor age ranged from 30 to 75 years. Collection was conducted in accordance with local ethics requirements, as described in detail previously [25]. 

The cells were cultured using reagents from Thermo Fisher Scientific (Thermo Fisher Scientific, Waltham, MA, USA) and culture plastic from Corning (Corning, NY, USA). MTT (3-(4,5-dimethylthiazol-2-yl)-2,5-diphenyl tetrazolium bromide) was produced by the company “PanEco” (PanEco, Moscow, Russian Federation). All reagents for high-performance liquid chromatography (HPLC) were purchased from Sigma-Aldrich (St. Louis, MO, USA) and Fluka (Buchs, Switzerland). 

### 4.2. Isolation of LGs 

LGs were isolated from the RPE of 85 cadaver eyes from donors aged 30–75 years, as described previously [14]. The initial LG concentration was 4 × 10^9^ granules/mL. The LG concentration was calculated in an N.K. Goryaev chamber. Then, suspension was divided into two samples of equal volume. Two types of LGs were obtained for the experiment. The first type of LGs was native unirradiated LGs. The second one was LGs irradiated for 10 min with visible light (400–700 nm) from a KGM 24–150 lamp (280 W/m^2^) measured with a photometer (Spectra-Physics 407A, Milpitas, CA, USA), with a heat filter at room temperature and constant stirring. The content of BisRets-OX was controlled with high-performance liquid chromatography (HPLC) (see below).

All stages of sample preparation were conducted under subdued lighting. 

### 4.3. High-Performance Liquid Chromatography (HPLC)

For the chromatographic analysis, chloroform extracts from LG suspensions were prepared using the Folch method [71], followed by drying and subsequently resuspending in 200 µL methanol, as described previously [44]. Each sample was passed through a Knauer chromatograph system (Germany) equipped with a Kromasil-100-5-C18 (4 × 250 mm; sorbent size, 5 μm) column, followed by HPLC, using a reverse phase gradient from 80% acetonitrile/20% water (vol/vol) (+0.05% TFA) to 100% acetonitrile for 20 min at a flow rate of 1.0 mL min^−1^, as described previously [44]. The HPLC analysis (K-2501 detector, Knauer) was performed at the wavelength of 430 nm. Precision for each sample was determined from two separately measured chromatograms for each individual sample at each wavelength.

### 4.4. A2E Synthesis

A2E as a standard was prepared from all-*trans* retinal and ethanolamine in acetic acid and ethanol, as described previously [72]. A2E was identified using a 7T LTQ FT mass spectrometer (Thermo Electron Corp., Bremen, Germany) equipped with an electrospray ion source, as described previously [32]. A2E purity was monitored with HPLC (see Section 4.3). The A2E concentration was determined spectrally using a Shimadzu UV-1700 spectrophotometer (Shimadzu, Kyoto, Japan) at a wavelength of 430 nm with *ε* = 3.1 × 10^4^ M^−1^cm^−1^.

### 4.5. Fluorescence Spectroscopy 

Fluorescence spectra were recorded using an RF-5301 PC fluorometer (Shimadzu, Kyoto, Japan) equipped with an R955 photomultiplier tube detector (Hamamatsu Photonics K.K., Hamamatsu City, Japan). Following excitation at 488 nm, emission spectra were obtained in the regions of 500–700 nm, at sampling intervals of 1 nm. Spectra were normalized to the wavelength of 592 nm [42]. 

### 4.6. ARPE-19 Cell Culture 

ARPE-19 cells were obtained from the Cell Culture Collection of the Koltzov Institute of Developmental Biology of Russian Academy of Sciences. RPE cell suspension at a concentration of 0.5 × 10^6^ (cells/dishes) was cultured in a supplemented culture media DMEM/F12 containing 10% of embryonic veal serum, 2 mM L-glutamine, and antibiotic–antimycotic (streptomycin, amphotericin B, penicillin) from Gibco (Thermo Fisher Scientific, Waltham, MA, USA). Cultivation was carried out in Petri dishes (d = 35 mm) under standard conditions (37 °C, 5% CO_2_) for 3 days. Thereafter, a change in complete nutrient medium was carried out. When the cells reached 70% of confluence, the nutrient medium was completely removed and 500 µL of accutase solution (Thermo Fisher Scientific, Waltham, Massachusetts, USA) was added, followed by incubation for 10 min. Then, the complete nutrient medium was added to the cell suspension to dilute the accutase 10 times. The resulting cell suspension was centrifuged (1100 rpm, 5 min, 37 °C). After centrifugation, the supernatant was removed and 1 mL of complete nutrient medium was added. To count the cells, the resulting suspension was placed in 10 µL disposable slides. An automatic counter of cells, Luna II (Logos biosystems, Anyang, Korea), was used.

### 4.7. Enriching ARPE-19 Cells with LGs 

The suspension of RPE cells at a concentration of 1.5 × 10^5^ (cells/dishes) was placed in Petri dishes (d = 35 mm) (Corning, NY, USA). Then, 1 mL of culture medium was added to the LG suspension, and 20 μL (per one Petri dish) of the diluted suspension (approximately 300 granules per cell) was added to a part of the Petri dishes with cell culture. Cultivation of cells with/without LGs was carried out 24 h in a complete nutrient medium (Thermo Fisher Scientific, Waltham, MA, USA), followed by a change in medium to remove nonphagocytic LGs. 

Two groups of samples were obtained, each of which consisted of a control sample (RPE cells without LGs), LG-fed RPE sample (original LGs) and LG*-fed RPE sample (LGs visible light irradiated). It should be noted that for all experiments, equal volumes from the same total suspension of LGs obtained from the RPE of 85 cadaver eyes were used.

The first group of samples was maintained in the dark. The second group of samples was irradiated for 18 h with visible light (430–570 nm) from a LED 15W-4000K lamp (0.38 mW/cm^2^). Subsequent analyses were performed simultaneously for both groups.

### 4.8. Cell Viability Assays

#### 4.8.1. The Live and Dead Assay

The Live and Dead assay stain solution was a mixture of two highly fluorescent dyes that differentially labelled live and dead cells. The Live and Dead cell assay was a one-step staining procedure. It could be used directly in cell culture media. 

For qualitative and quantitative analyses, all ARPE-19 cell samples were stained with the fluorescent dye Live and Dead (ab 115347 Abcam, Cambridge, UK) according to the protocol presented by the manufacturer. For the qualitative analysis, the cell culture was washed three times with phosphate buffer solution (PanEco, Moscow, Russian) and fixed in 10% formalin (PanEco, Moscow, Russian) for 20 min. Incorporation of LGs was monitored with a confocal microscope (Olympus Fluoview FV-10i, Tokyo, Japan) using the green channel AF 488 (Ex 490 nm/Em 525 nm) and red channel AF 594 (Ex 590 nm/Em 617 nm). 

For the quantitative analysis, the RPE cell suspension was obtained with the enzymatic removal of cells from the culture plastic, washed three times in CellWash solution (BD, Franklin Lakes, NJ, USA), and analyzed with a flow cytofluorometer (CytoFlex, Beckman Coulter, Brea, CA, USA) using the detection channels FITC (Ex 493 nm/Em 528 nm) and Cy3 (Ex 550 nm/Em 615 nm).

#### 4.8.2. MTT Reduction

The assessment with MTT assay was performed according to the protocol (https://www.abcam.com/mtt-assay-kit-cell-proliferation-ab211091.html (accessed on 16 June 2021)). For this assay, 4 × 96-well plates (Corning, NY, USA) were used. An amount of 200 μL of culture medium containing 3 × 10^3^ RPE cells were added to each well. Cultivation was carried out for 24 h under standard conditions. Furthermore, 1 µL of LG suspension was added to each well of the corresponding groups. Cultivation of cells with LGs was carried out for 24 h, after which the growth medium was replaced, and light exposure was performed for 18 h. After the RPE cells were exposed to the experimental conditions, the medium was removed. An amount of 300 µL of MTT (0.25 mG/mL in PBS (Thermo Fisher Scientific, Waltham, MA, USA)) was added to each well. The cells were incubated for 30 min at 37 °C in a humidified 5% CO_2_ incubator. The unreacted MTT was aspirated off and 300 µL of isopropanol-2 (Sigma-Aldrich, St louis, USA) was added to solubilize the reduced formazan crystals. After a 10 min incubation of the samples, the optical density was measured at 570 nm with a spectrophotometer Multiskan GO (Thermo Fisher Scientific, Waltham, ma, USA). Absorbances were normalized against the control sample (RPE cells without LGs) absorbance and were expressed as the viability as a percentage of control absorbance.

### 4.9. Statistical Analysis

GraphPad™ Prism 6 software (GraphPad Software Inc., La Jolla, CA, USA) was used to generate graphs and to calculate levels of significance. Levels of significance were calculated using the dispersion analysis (two-way ANOVA). Probability values of *p* < 0.05 were considered as statistically significant. 

## 5. Conclusions

We showed that LG BisRets can exhibit not only phototoxic properties, being ROS generators, but can also exhibit cytotoxicity after their oxidation by these ROS. As we have shown [38], as a result of photooxidation of BisRets in LGs, hydrophilic and amphiphilic carbonyl compounds are formed in the free state, capable of diffusing from LGs to the cytoplasmic part of the RPE cell. We have also shown [41] that these compounds can modify proteins, which, in turn, can cause carbonyl stress [34,35]. 

In this work, we demonstrated that LGs could have a damaging effect on the RPE cell through the BisRets-OX formation. These products could be matched to the formation of lipid peroxidation end products, i.e., highly reactive electrophilic aldehydes such as malondialdehyde (MDA) and 4-hydroxynonenal (HNE) in LGs, which suggests a connection between its formation and increased oxidative stress [73,74,75,76]. 

Thus, the data obtained showed that the effects of oxidative stress in the RPE cell are long-term, including due to the cytotoxic BisRets-OX formation inside LGs when exposed to light.

## Figures and Tables

**Table 1:** Comparative flow cytofluorimetry analysis of cell viability when incubating RPE samples in the dark or after irradiation of RPE samples with visible light.

Time of Dark Incubation	First Group			Time of Dark Incubation	Second Group		
	Samples Maintained in the Dark (%)				Samples Irradiated with Visible Light for 18 h (%)		
	Control RPE without LGs	LG-fed RPE	LG*-fed RPE		Control RPE without LGs	LG-fed RPE	LG*-fed RPE
18 h	93.67 ± 1.84	90.88 ± 0.59	90.53 ± 0.59	0 days	93.41 ± 0.46	90.54 ± 0.93	90.39 ± 0.54
4 days	82.82 ± 2.47	70.66 ± 6.39	70.47 ± 1.12	4 days	66.73 ± 3.96	39.84 ± 3.49	48.87 ± 1.23

Data demonstrate the relative content of live RPE cells under these experimental conditions. Data are presented as means ± SDs from three independent experiments.

**Table 2:** MTT cell viability assay when RPE samples were maintained in the dark or after irradiation of RPE samples with visible light.

Time of Dark Incubation	First Group			Time of Dark Incubation	Second Group		
	Samples Maintained in the Dark (%)				Samples Irradiated with Visible Light for 18 h (%)		
	Control RPE without LGs	LG-fed RPE	LG*-fed RPE		Control RPE without LGs	LG-fed RPE	LG*-fed RPE
18 h	100.0 ± 28.3	71.8 ± 16.0	75.2 ± 21.9	0 days	100.0 ± 33.4	67.8 ± 16.4	66.5 ± 24.3
4 days	100.0 ± 24.9	77.4 ± 17.8	83.9 ± 24.7	4 days	100.0 ± 15.3	53.8 ± 15.9	67.9 ± 14.0

Data demonstrate the relative content of living RPE cells regarding the control sample of RPE cells without LGs under these experimental conditions. Data are presented as means ± SDs from three independent experiments.

## Data Availability

The data presented in this study are available in the article and on request from the corresponding author.

## References

[1] Ptito M., Bleau M., Bouskila J. (2021). The Retina: A Window into the Brain. Cells.

[2] Fisher C.R., Ferrington D.A. (2018). Perspective on AMD pathobiology: A bioenergetic crisis in the RPE. Investig. Ophthalmol. Vis. Sci..

[3] Abokyi S., To C.-H., Lam T.T., Tse D.Y. (2020). Central role of oxidative stress in age-related macular degeneration: Evidence from a review of the molecular mechanisms and animal models. Oxidative Med. Cell Longev..

[4] Beatty S., Koh H.-H., Phil M., Henson D., Boulton M. (2000). The Role of Oxidative Stress in the Pathogenesis of Age-Related Macular Degeneration. Surv. Ophthalmol..

[5] Kohen R., Nyska A. (2002). Invited Review: Oxidation of biological systems: Oxidative stress phenomena, antioxidants, redox reactions, and methods for their quantification. Toxicol. Pathol..

[6] Strauss O. (2005). The retinal pigment epithelium in visual function. Physiol. Rev..

[7] Wolf G. (2003). Lipofuscin and macular degeneration. Nutr. Rev..

[8] Young R.W. (1967). The renewal of the photoreceptor cell outer segments. J. Cell Biol..

[9] Kennedy C.J., Rakoczy P.E., Constable I.J. (1995). Lipofuscin of the retinal pigment epithelium: A review. Eye.

[10] Feeney L. (1973). The phagosomal system of the pigment epithelium: A key to retinal disease. Investig. Ophthalmol. Vis. Sci..

[11] Feeney-Burns L., Hilderbrand E.S., Eldridge S. (1984). Aging human RPE: Morphometric analysis of macular, equatorial, and peripheral cells. Investig. Ophthalmol. Vis. Sci..

[12] Jung T., Bader N., Grune T. (2007). Lipofuscin. Formation, distribution, and metabolic consequences. Ann. N. Y. Acad. Sci..

[13] Yin D. (1996). Biochemical basis of lipofuscin, ceroid, and age pigment-like fluorophores. Free Rad. Biol. Med..

[14] Boulton M., Dontsov A., Jarvis-Evans J., Ostrovsky M., Svistunenko D. (1993). Lipofuscin is a photoinducible free radical generator. J. Photochem. Photobiol. B Biol..

[15] Holz F.G., Pauleikhoff D., Klein R., Bird A.C. (2004). Pathogenesis of lesions in late age-related macular disease. Am. J. Ophthalmol..

[16] Katz M.L. (2002). Potential role of retinal pigment epithelial lipofuscin accumulation in age-related macular degeneration. Arch. Gerontol. Geriatrics.

[17] Sparrow J.R., Boulton M.E. (2005). RPE lipofuscin and its role in retinal pathobiology. Exp. Eye Res..

[18] Lamb L.E., Simon J.D. (2004). A2E: A component of ocular lipofuscin. Photochem. Photobiol..

[19] Sakai N., Decatur J., Nakanishi K., Eldred G.E. (1996). Ocular age pigment “A2E”: An unprecedented pyridinium bisretinoid. J. Am. Chem. Soc..

[20] Sparrow J.R., Kim S.R., Cuervo A.M., Bandhyopadhyayand U. (2008). A2E, a pigment of RPE lipofuscin, is generated from the precursor, A2PE by a lysosomal enzyme activity. Adv. Exp. Med. Biol..

[21] Sparrow J.R., Gregory-Roberts E., Yamamoto K., Blonska A., Ghosh S.K., Ueda K., Zhou J. (2012). The bisretinoids of retinal pigment epithelium. Prog. Retin. Eye Res..

[22] Wang Z., Keller L.M.M., Dillon J., Gaillard E.R. (2006). Oxidation of A2E results in the formation of highly reactive aldehydes and ketones. Photochem. Photobiol..

[23] Wu Y., Yanase E., Feng X., Siegel M.M., Sparrow J.R. (2010). Structural characterization of bisretinoid A2E photocleavage products and implications for age-related macular degeneration. Proc. Natl. Acad. Sci. USA.

[24] Kim S.R., Jang Y.P., Jockusch S., Fishkin N.E., Turro N.J., Sparrow J.R. (2007). The all-trans-retinal dimer series of lipofuscin pigments in retinal pigment epithelial cells in a recessive Stargardt disease model. Proc. Natl. Acad. Sci. USA.

[25] Feldman T.B., Yakovleva M.A., Arbukhanova P.M., Borzenok S.A., Kononikhin A.S., Popov I.A., Nikolaev E.N., Ostrovsky M.A. (2015). Changes in spectral properties and composition of lipofuscin fluorophores from human retinal pigment epithelium with age and pathology. Anal. Bioanal. Chem..

[26] Sparrow J.R., Nakanishi K., Parish C.A. (2000). The lipofuscin fluorophore A2E mediates blue light-induced damage to retinal pigment epithelial cells. Investig. Ophthalmol. Vis. Sci..

[27] Rozanowska M., Jarvis-Evans J., Korytowski W., Boulton M.E., Burke J.M., Sarna T. (1995). Blue light-induced reactivity of retinal age pigment. In vitro generation of oxygen-reactive species. J. Biol. Chem..

[28] Rozanowska M., Wessels J., Boulton M., Burke J.M., Rodgers M.A.J., Truscott T.G., Sarna T. (1998). Blue light-induced singlet oxygen generation by retinal lipofuscin in non-polar media. Free Radic. Biol. Med..

[29] Różanowska M., Sarna T. (2005). Light-induced damage to the retina: Role of rhodopsin chromophore revisited. Photochem. Photobiol..

[30] Avalle L.B., Dillon J., Tari S., Gaillard E.R. (2005). A new approach to measuring the action spectrum for singlet oxygen production by human retinal lipofuscin. Photochem. Photobiol..

[31] Ben-Shabat S., Itagaki Y., Jockusch S., Sparrow J.R., Turro N.J., Nakanishi K. (2002). Formation of a nona-oxirane from A2E, a lipofuscin fluorophore related to macular degeneration, and evidence of singlet oxygen involvement. Angew. Chem. Int. Ed. Engl..

[32] Yakovleva M.A., Sakina N.L., Kononikhin A.S., Feldman T.B., Nikolaev E.N., Dontsov A.E., Ostrovsky M.A. (2006). Detection and study of the products of photooxidation of N-Retinylidene-N-retinylethanolamine (A2E), the fluorophore of lipofuscin granules from retinal pigment epithelium of human donor eyes. Dokl. Biochem. Biophys..

[33] Yoon K.D., Yamamoto K., Ueda K., Zhou J., Sparrow J.R. (2012). A novel source of methylglyoxal and glyoxal in retina: Implications for age-related macular degeneration. PLoS ONE.

[34] Ergin V., Ebrahimi R., Karasu C. (2013). Carbonyl stress in aging process: Role of vitamins and phytochemicals as redox regulators. Aging Dis..

[35] Schleicher E.D., Bierhaus A., Haring H.U., Nawroth P.P., Lehmann R. (2001). Chemistry and pathobiology of advanced glycation end products. Contrib. Nephrol..

[36] Dontsov A.E., Sakina N.L., Golubkov A.M., Ostrovsky M.A. (2009). Light-induced release of A2E photooxidation toxic products from lipofuscin granules of human retinal pigment epithelium. Dokl. Biochem. Biophys..

[37] Sparrow J.R., Vollmer-Snarr H.R., Zhou J., Jang Y.P., Jockusch S., Itagaki Y., Nakanishi K. (2003). A2E-epoxides damage DNA in retinal pigment epithelial cells. Vitamin E and other antioxidants inhibit A2E-epoxide formation. J. Biol. Chem..

[38] Yakovleva M., Dontsov A., Trofimova N., Sakina N., Kononikhin A., Aybush A., Gulin A., Feldman T., Ostrovsky M. (2022). Lipofuscin granule bisretinoid oxidation in the human retinal pigment epithelium forms cytotoxic carbonyls. Int. J. Mol. Sci..

[39] Munch G., Schicktanz D., Behme A., Gerlach M., Riederer P., Palm D., Schinzel R. (1999). Amino acid specificity of glycation and protein-AGE crosslinking reactivities determined with a dipeptide SPOT library. Nat. Biotechnol..

[40] Ott C., Jacobs K., Haucke E., Navarrete Santos A., Grune T., Simm A. (2014). Role of advanced glycation end products in cellular signaling. Redox. Biol..

[41] Dontsov A., Yakovleva M., Trofimova N., Sakina N., Gulin A., Aybush A., Gostev F., Vasin A., Feldman T., Ostrovsky M. (2022). Water-soluble products of photooxidative destruction of the bisretinoid A2E cause proteins modification in the dark. Int. J. Mol. Sci..

[42] Feldman T.B., Yakovleva M.A., Larichev A.V., Arbukhanova P.M., Radchenko A.S., Borzenok S.A., Kuzmin V.A., Ostrovsky M.A. (2018). Spectral analysis of fundus autofluorescence pattern as a tool to detect early stages of degeneration in the retina and retinal pigment epithelium. Eye.

[43] Feldman T.B., Yakovleva M.A., Dontsov A.E., Ostrovsky M.A. (2010). Fluorescence emission and excitation spectra of fluoro-phores of lipofuscin granules isolated from retinal pigment epithelium of human cadaver eyes. Russ. Chem. Bull. Int. Ed..

[44] Yakovleva M.A., Radchenko A.S., Feldman T.B., Kostyukov A.A., Arbukhanova P.M., Borzenok S.A., Kuzmin V.A., Ostrovsky M.A. (2020). Fluorescence characteristics of lipofuscin fluorophores from human retinal pigment epithelium. Photochem. Photobiol. Sci..

[45] Schweitzer D., Schenke S., Hammer M., Schweitzer F., Jentsch S., Birckner E., Becker W., Bergmann A. (2007). Towards metabolic mapping of the human retina. Microsc. Res. Tech..

[46] Mosmann T. (1983). Rapid colorimetric assay for cellular growth and survival: Application to proliferation and cytotoxicity assays. J. Immunol. Methods.

[47] Godley B.F., Shamsi F.A., Liang F.-Q., Jarrett S.G., Davies S., Boulton M. (2005). Blue light induces mitochondrial DNA damage and free radical production in epithelial cells. J. Biol. Chem..

[48] Surin A.M., Sharipov R.R., Krasil’nikova I.A., Boyarkin D.P., Lisina O.Y., Gorbacheva L.R., Avetisyan A.V., Pinelis V.G. (2017). Disruption of functional activity of mitochondria during MTT assay of viability of cultured neurons. Biochemistry.

[49] Shamsi F.A., Boulton M. (2001). Inhibition of RPE lysosomal and antioxidant activity by the age pigment lipofuscin. Investig. Ophthalmol. Vis. Sci..

[50] Alaimo A., Liñares G.G., Bujjamer J.M., Gorojod R.M., Alcon S.P., Martínez J.H., Baldessari A., Grecco H.E., Kotler M.L. (2019). Toxicity of blue led light and A2E is associated to mitochondrial dynamics impairment in ARPE-19 cells: Implications for age-related macular degeneration. Arch. Toxicol..

[51] Schütt F., Davies S., Kopitz J., Boulton M., Holz F.G. (2000). A retinoid constituent of lipofuscin, A2E is a photosensitizer in hu-man retinal pigment epithelial cells. Ophthalmologe.

[52] Murdaugh L.S., Avalle L.B., Mandal S., Dill A.E., Dillon J., Simon J.D., Gaillard E.R. (2010). Compositional studies of human RPE lipofuscin. J. Mass. Spectrom..

[53] Murdaugh L.S., Mandal S., Dill A.E., Dillon J., Simon J.D., Gaillard E.R. (2011). Compositional studies of human RPE lipofuscin: Mechanisms of molecular modifications. J. Mass. Spectrom..

[54] Zhou J., Jang Y., Kim S., Sparrow J. (2006). Complement activation by photooxidation products of A2E, a lipofuscin constituent of the retinal pigment epithelium. Proc. Natl. Acad. Sci. USA.

[55] Khansari N., Shakiba Y., Mahmoudi M. (2009). Chronic inflammation and oxidative stress as a major cause of age-related diseases and cancer. Recent Pat. Inflamm. Allergy Drug Discov..

[56] Johnson L., Leitner W., Staples M., Anderson D. (2001). Complement activation and inflammatory processes in drusen formation and age-related macular degeneration. Exp. Eye Res..

[57] Anderson D.H., Mullins R.F., Hageman G.S., Johnson L.V. (2002). A role for local inflammation in the formation of drusen in the aging eye. Am. J. Ophthalmol..

[58] Despriet D.D., van Duijn C.M., Oostra B.A., Uitterlinden A.G., Hofman A., Wright A.F., Jacoline B., Bakker A., de Jong P.T., Vingerling J.R. (2009). Complement component C3 and risk of age-related macular degeneration. Ophthalmology.

[59] Hollyfield J.G. (2010). Age-related macular degeneration: The molecular link between oxidative damage, tissue-specific inflammation and outer retinal disease: The Proctor Lecture. Investig. Ophthalmol. Vis. Sci..

[60] Kauppinen A., Paterno J.J., Blasiak J., Salminen A., Kaarniranta K. (2016). Inflammation and its role in age-related macular degeneration. Cell Mol. Life Sci..

[61] Crabb J.W., Miyagi M., Gu X., Shadrach K., West K.A., Sakaguchi H., Kamei M., Hasan A., Yan L., Rayborn M.E. (2002). Drusen proteome analysis: An approach to the etiology of age-related macular degeneration. Proc. Natl. Acad. Sci. USA.

[62] Hollyfield J.G., Crabb J.W., Salomon R.G. (2003). Proteomic approaches to understanding age-related macular degeneration. Adv. Exp. Med. Biol..

[63] Wiktor A., Sarna M., Wnuk D., Sarna T. (2018). Lipofuscin-mediated photodynamic stress induces adverse changes in nanomechanical properties of retinal pigment epithelium cells. Sci. Rep..

[64] Yakovleva M.A., Feldman T.B., Lyakhova K.N., Utina D.M., Kolesnikova I.A., Vinogradova Y.V., Molokanov A.G., Ostrovsky M.A. (2022). Ionized radiation-mediated retinoid oxidation in the retina and retinal pigment epithelium of the murine eye. Radiat. Res..

[65] Kobashigawa S., Suzuki K., Yamashita S. (2011). Ionizing radiation accelerates Drp1-dependent mitochondrial fission, which involves delayed mitochondrial reactive oxygen species production in normal human fibroblast-like cells. Biochem. Biophys. Res. Commun..

[66] Chien T., Tseng T.L., Wang J.Y., Shen Y.T., Lin T.H., Shieh J.C. (2015). Candida albicans DBF4 gene inducibly duplicated by the mini-Urablaster is involved in hypha-suppression. Mutat. Res..

[67] Tisi A., Feligioni M., Passacantando M., Ciancaglini M., Maccarone R. (2021). The impact of oxidative stress on blood-retinal barrier physiology in age-related macular degeneration. Cells.

[68] Olchawa M.M., Furso J.A., Szewczyk G.M., Sarna T.J. (2017). Lipofuscin-mediated photic stress inhibits phagocytic activity of ARPE-19 cells; effect of donors’ age and antioxidants. Free Radic. Res..

[69] Totan Y., Yağci R., Bardak Y., Ozyurt H., Kendir F., Yilmaz G., Sahin S., Sahin T.U. (2009). Oxidative macromolecular damage in age-related macular degeneration. Curr. Eye Res..

[70] Lu L., Gu X., Hong L., Laird J., Jaffe K., Choi J., Crabb J., Salomon R.G. (2009). Synthesis and structural characterization of carboxyethylpyrrole-modified proteins: Mediators of age-related macular degeneration. Bioorg. Med. Chem..

[71] Folch J., Lees M., Stanley G.H.S. (1957). A simple method for the isolation and purification of total lipids from animal tissues. J. Biol. Chem..

[72] Parish C.A., Hashimoto M., Nakanishi K., Dillon J., Sparrow J. (1998). Isolation and one-step preparation of A2E and iso-A2E, fluorophores from human retinal pigment epithelium. Proc. Natl. Acad. Sci. USA.

[73] Schutt F., Bergmann M., Holz F.G., Kopitz J. (2003). Proteins modified by malondialdehyde,4-hydroxynonenal, or advanced glycation end products in lipofuscin of human retinal pigment epithelium. Investig. Ophthalmol. Vis. Sci..

[74] Ye F., Kaneko H., Hayashi Y., Takayama K., Hwang S.J., Nishizawa Y., Kimoto R., Nagasaka Y., Tsunekawa T., Matsuura T. (2016). Malondialdehyde induces autophagy dysfunction and VEGF secretion in the retinal pigment epithelium in age-related macular degeneration. Free Radic. Biol. Med..

[75] Hyttinen J.M.T., Viiri J., Kaarniranta K., Błasiak J. (2018). Mitochondrial quality control in AMD: Does mitophagy play a pivotal role?. Cell Mol. Life Sci..

[76] Rózanowska M.B., Rózanowski B. (2022). Photodegradation of lipofuscin in suspension and in ARPE-19 cells and the similarity of fluorescence of the photodegradation product with oxidized docosahexaenoate. Int. J. Mol. Sci..

